# Murder of Innocents

**Published:** 1871-04

**Authors:** 


### MURDER OF INNOCENTS.

A LONG, loud wail of intense objurgation comes from New England, the land of school-houses and churches, of great ideas and progress, on account of the sentiments expressed on page 68 of the February number of the JOURNAL. It is a relief to know that it is from a man, not from a woman.

From the most populous part of that same New England, from the region round about the Hub, information comes that more than two thirds of all the living children are born of foreigners; in other words are Roman Catholic born. Three out of four letters coming to city physicians wanting information as to the safest and best means of nipping the flower in the bud, of blighting an immortal life, come from educated, enterprising, thrifty New England Protestants, never a one from a Roman Catholic; these latter are taught early, taught plainly, that the attempt to blight an immortal nature is murder, even if it does not succeed. It is murder, if the thought is in the heart previous to a prolific union. Protestants are too modest to put forth any teachings on the subject; hence the arch adversary has nothing to do but sow the seed of "worldly prudence;" and these, watered and manured by considerations of worldly wisdom, grow up into practices more hurtful and wicked, more pernicious to the body, more degrading to the morals, than practices with similar results which prevail among

Eastern nations of a lower civilization. Certainly the great difference between the number of the children of American and foreign parentage in and around Boston, where the population is about half and half, does not all arise from the practices referred to, nor largely, but only in a small part; still to that extent the subject is worthy of notice with reprobation, lest silence might lead to its extension.

